# Efficient Convolutional Neural Network-Based Keystroke Dynamics for Boosting User Authentication

**DOI:** 10.3390/s23104898

**Published:** 2023-05-19

**Authors:** Hussien AbdelRaouf, Samia Allaoua Chelloug, Ammar Muthanna, Noura Semary, Khalid Amin, Mina Ibrahim

**Affiliations:** 1 Department of Information Technology, Faculty of Computers and Information, Menoufia University, Shebin El-Kom 32511, Menoufia, Egypt; noura.semary@ci.menofia.edu.eg (N.S.); k.amin@ci.menofia.edu.eg (K.A.); mina.ibrahim@ci.menofia.edu.eg (M.I.); 2 Department of Information Technology, College of Computer and Information Sciences, Princess Nourah bint Abdulrahman University, P.O. Box 84428, Riyadh 11671, Saudi Arabia; 3 Department of Applied Probability and Informatics, RUDN University, 6 Miklukho-Maklaya St, Moscow 117198, Russia; ammarexpress@gmail.com

**Keywords:** keystroke dynamics, user authentication, convolutional neural network, CNN, CMU, quantile transformation, deep learning, boosting techniques

## Abstract

The safeguarding of online services and prevention of unauthorized access by hackers rely heavily on user authentication, which is considered a crucial aspect of security. Currently, multi-factor authentication is used by enterprises to enhance security by integrating multiple verification methods rather than relying on a single method of authentication, which is considered less secure. Keystroke dynamics is a behavioral characteristic used to evaluate an individual’s typing patterns to verify their legitimacy. This technique is preferred because the acquisition of such data is a simple process that does not require any additional user effort or equipment during the authentication process. This study proposes an optimized convolutional neural network that is designed to extract improved features by utilizing data synthesization and quantile transformation to maximize results. Additionally, an ensemble learning technique is used as the main algorithm for the training and testing phases. A publicly available benchmark dataset from Carnegie Mellon University (CMU) was utilized to evaluate the proposed method, achieving an average accuracy of 99.95%, an average equal error rate (EER) of 0.65%, and an average area under the curve (AUC) of 99.99%, surpassing recent advancements made on the CMU dataset.

## 1. Introduction

The process of confirming users’ identities is a crucial aspect of digital systems. Companies and corporations are actively seeking effective security solutions to address threats, including identity theft and data leaks. The COVID-19 pandemic has underscored the significance of ensuring secure authentication when accessing business and personal information over the Internet. The ability to work from home and perform regular tasks online, such as completing insurance paperwork and making purchase orders, has become increasingly important. Unfortunately, recent events have also led to a rise in security breaches [1].

Biometrics refers to an individual’s characteristics or actions and can be classified into physiological and behavioral categories [2]. The behavioral biometric technique of keystroke dynamics utilizes a person’s typing patterns on a keyboard. Keystroke dynamics have significant advantages due to their ability to identify individuals based on their unique typing rhythm. The significant advantages of keystroke dynamics are:

- **Uniqueness:** According to [3], keystroke inputs can be accurately measured using software, which makes it extremely difficult to reproduce someone’s typing patterns at the same level of precision without a significant amount of effort.
- **Low Cost:** Unlike other biometric systems that rely on physical hardware, such as face or fingerprint recognition, keystroke dynamics can be implemented solely through software. This approach reduces installation costs and makes it suitable for remote authentication [4,5]. This has led many service providers to develop their own methods or utilize third-party solutions to validate their users’ credentials [6].
- **Enhances Password Longevity and Robustness:** Passwords remain the most widely used form of authentication, despite their vulnerability. Keystroke dynamics is being explored as a method to enhance the security of passwords and increase their durability [4].
- **Ongoing Verification and Monitoring:** Keystroke dynamics offer a way to continually authenticate a person’s legal identity as long as they continue to communicate with the system using input keyboards [7], as it is possible to continuously analyze and reassess keystroke typing behavior.

There are two primary kinds of keystroke dynamics, free text and fixed text. Free text concentrates on authenticating a user’s identity using impromptu text, which typically requires extensive training and large text instances. On the other hand, fixed text aims to confirm a user’s identity based on repeated, short text and requires a much shorter training period. Fixed text methods are commonly used by service providers to verify a user’s identity as they enter their login credentials, offering clear benefits. These methods also help prevent identity theft, fraud, and cyberattacks, thereby increasing security without requiring any extra work or action on the part of the user. Keystroke dynamics is becoming increasingly popular as a means of enhancing the security of Internet of Things (IoT) devices. One key advantage of keystroke dynamics is that it does not require any additional hardware or software to be installed on a device. It can be implemented using existing keyboard hardware and software, making it a cost-effective solution for IoT devices compared to cryptography and hash functions that require a lot of computation [8,9,10].

In this paper, we will focus on meeting two essential criteria:

- **Performance:** We aim to enhance the recognition accuracy of keystroke dynamics, surpassing that of previous research.
- **Robustness:** The most crucial factor is to ensure that the suggested method can effectively handle issues such as overfitting, underfitting, noise, and outliers.

Developing a technique that fulfills these criteria is a difficult task. To address this issue, this study proposes an effective method that relies on advanced preprocessing for reducing outliers, a tailored convolutional neural network for extracting the best features, and a boosting technique for accurately detecting an attacker or normal user.

Table 1 provides a summary of various recent research studies aimed at improving and developing keystroke dynamics authentication. In this paper, we propose a straightforward technique that fulfills the above-mentioned criteria by utilizing an effective preprocessing technique, a deep learning architecture, and a boosting classifier to enhance user authentication.

The most significant contributions of our study are:

- Compared to other biometrics, keystroke dynamics offers certain benefits, but its main drawback is its lower accuracy. This research aims to address this limitation by investigating the performance and limitations of existing systems and proposing a model that can enhance the accuracy and performance of biometric systems.
- A novel data synthesization technique that effectively augments and increases the data using the standard deviation.
- Reduction of anomalies and extreme values in the data through the use of quantile transformation, which converts any distribution into a uniform distribution.
- An efficient, tailored convolutional neural network that is robust to overfitting and underfitting problems.
- Enhanced performance and robustness using a combination of data synthesization and quantile transformation techniques.

The proposed methodology in this study further optimizes the use of keystroke dynamics for authentication by utilizing an optimized convolutional neural network that extracts improved features, together with an ensemble learning technique for the training and testing phases. The overall advantage of this methodology is that it enhances the security of online services by providing a highly accurate and efficient authentication process that utilizes a unique and convenient method of user identification.

The remaining sections of our paper are organized as follows. Section 2 describes the previous research that is relevant to our study. In Section 3, we provide a detailed discussion of the proposed method, including the preprocessing and learning algorithms used. The experimental results, including the dataset, evaluation metrics, and outcomes, are described in detail in Section 4. Finally, in Section 5, we summarize our findings and suggest potential areas for further investigation.

## 2. Related Works

In this section, we examine the recent works that have applied machine learning methods and then explore deep learning approaches.

### 2.1. Machine Learning Techniques

Recently, machine learning methods have been extensively employed in research on keystroke dynamics.

In [11], the main classifier used in the training and testing phase was Histogram Gradient Boosting (HGB), which relies on histograms to group continuous data into a fixed number of bins. By reducing the number of unique values for each feature to a smaller, more manageable set, this technique allows for faster and more efficient implementation of decision trees. This approach achieved an average accuracy of 97.96%, which is comparable to that of traditional gradient boosting.

Another study [12] employed multiple machine learning techniques, including XGBoost, a popular method in Kaggle competitions known for its ability to handle outliers and misclassifications better than AdaBoost. Data augmentation was also used, which involves generating synthetic data to supplement an existing dataset. Through experiments, the authors found that XGBoost with data augmentation achieved the highest accuracy of 96.39%.

In [13], a novel barcoding system was proposed, which transforms biometric data into compact barcode images that can be easily stored. The primary technique utilized for training the barcode images was one-class SVM. The results of this approach were promising, with an excellent EER (Equal Error Rate) of 1.83%.

As the dimensionality of data increases, the data become sparse and all data points seem like outliers when using distance measures. To address this issue, a subspace-based algorithm was proposed in [14], which uses the sparsity coefficient for finding outliers. Partitioning high-dimensional space into subspaces results in a large number of subspaces, making it difficult to search through each one in a timely manner. To solve this issue, Modified Differential Evolution (MDE) is used to search for sparse subspaces by using the sparsity coefficient as the objective function. MDE’s crossover rate and mutation explore and exploit these sparse subspaces, achieving an equal error rate of 3.48%.

In [15], the limitations of keystroke dynamics algorithms when multiple users share an account were addressed. The authors introduced a four-stage approach that utilizes pre-existing algorithms to autonomously ascertain the number of users who share an account and provide reliable support for accounts that are shared by multiple users. The approach resulted in an average improvement of 9.2% for the AUC and 8.6% for the EER in cases where multiple users were involved. Several research studies have employed the K-nearest neighbor (KNN) method for user authentication. For example, KNN was used together with dependence clustering [16], resulting in an equal error rate (EER) of 7.7%. In another study, KNN was combined with dimensionality reduction and localization [17], which was found to be effective in handling anomalies and deviations with an accuracy of 87.5%. Another paper [18] proposed a novel method for keystroke dynamics-based authentication using the Generalized Fuzzy Model (GFM), which outperformed the Gaussian Mixture Model (GMM) on the CMU dataset. The best equal error rate (EER) of 7.86% was achieved with GFM using Hold Time and Up-Down Time with 16 components. GFM’s superiority over GMM was attributed to the use of uncertainty representation in keystroke measurements, which allows for good accuracy with few Gaussian mixture components.

Ali et al. [19] proposed a hybrid POHMM/SVM method for efficient user identification in keystroke biometrics. The POHMM model extracts features and handles missing or infrequent data, and SVM is used as the classifier. The proposed model achieved the best accuracy of 91.3% and inherited the benefits of both the POHMM and SVM models.

Benoît et al. suggested an authentication method based on a bagging ensemble of three different classifiers: SVM, KNN, and decision tree [20]. The method uses keystroke dynamics to authenticate users based on their typing style and achieved an accuracy of 95.65% on the CMU dataset. The proposed approach combines the outputs of the three classifiers using majority voting, resulting in better accuracy than previous works.

### 2.2. Deep Learning Techniques

Deep learning techniques have demonstrated exceptional performance in classification tasks across various fields, including keystroke dynamics. By training deep neural networks on keystroke data, researchers have been able to automatically extract features and patterns that distinguish individual typing behaviors, such as keystroke timing, duration, and pressure. This has enabled the development of more accurate and reliable keystroke dynamics authentication systems, which can effectively identify individuals based on their typing patterns.

Adesh et al. [21] analyzed a keystroke dataset using SVM, RF, and ANN, with the latter achieving the best accuracy of 91.8%. The ANN had 6 layers and 56 output nodes, with various activation functions and dropout regularization used. The authors of [22] proposed a deep learning approach using three different neural network models trained for each user. Resilient backpropagation with a momentum factor was used to reduce weight change fluctuations. The 20-30-20 neural network configuration provided the best results, with an ERR of 0.049 and identification accuracy of 94.7%, outperforming models with more than three hidden layers. In [23], transfer learning was proposed, which converts keystroke data into image data and applies fine-tuning to pre-trained AlexNet and ResNet models for classification. The authors also used a support vector machine for feature extraction. The approach achieved 98.57% accuracy. An autoencoder model with encoder and decoder phases was utilized for keystroke authentication [24]. An MLP architecture was used in the encoder phase to obtain features, and the decoder phase reconstructed the original features using the extracted features. The encoded features with minimum errors were fed into a Gaussian mixture algorithm to determine abnormal users, achieving an EER of 6.51.

The use of various optimizers in deep learning approaches has shown significant improvements in performance. Muliono et al. [25] employed several independent learning layers that can learn separately. Their research highlighted the significance of using the Nadam optimizer, which demonstrated an impressive accuracy of 92.60%. To speed up the training process of deep networks, researchers frequently utilize various optimizers, in particular, the Adam optimizer [26]. The authors of [26] proposed a three-hidden-layer architecture utilizing LeakyReLU and softmax functions, achieving an overall accuracy of 93.59% and an EER of 3%. Andrean et al. [27] proposed a Multilayer Perceptron (MLP)-based deep learning approach for keystroke authentication. The MLP architecture has an input layer, two hidden layers, and an output layer. The approach achieved an optimal EER of 4.45% using 31 input neurons and 23 hidden neurons. Table 1 provides an overview of the most recent advances made in the CMU dataset utilizing both machine and deep learning techniques.

It is evident from the literature that researchers mainly use one of two approaches. The first approach involves the use of various machine learning algorithms to improve classification performance and the second approach involves the utilization of deep learning approaches to extract features and enhance performance. In our study, we aim to develop a new approach that combines the benefits of both these approaches by employing a customized deep learning architecture to extract optimal features and using boosting techniques to classify the features into the correct category and identify malicious users. In addition to combining the advantages of both approaches, we also employ two efficient data processing techniques to improve performance. The first is data synthesization to increase the amount of data and the second is quantile transformation to effectively reduce the impact of outliers. As stated in the literature, most studies focus on improving the EER and accuracy, which still require further advancement and refinement.

## 3. Methodology

**Figure 1:**
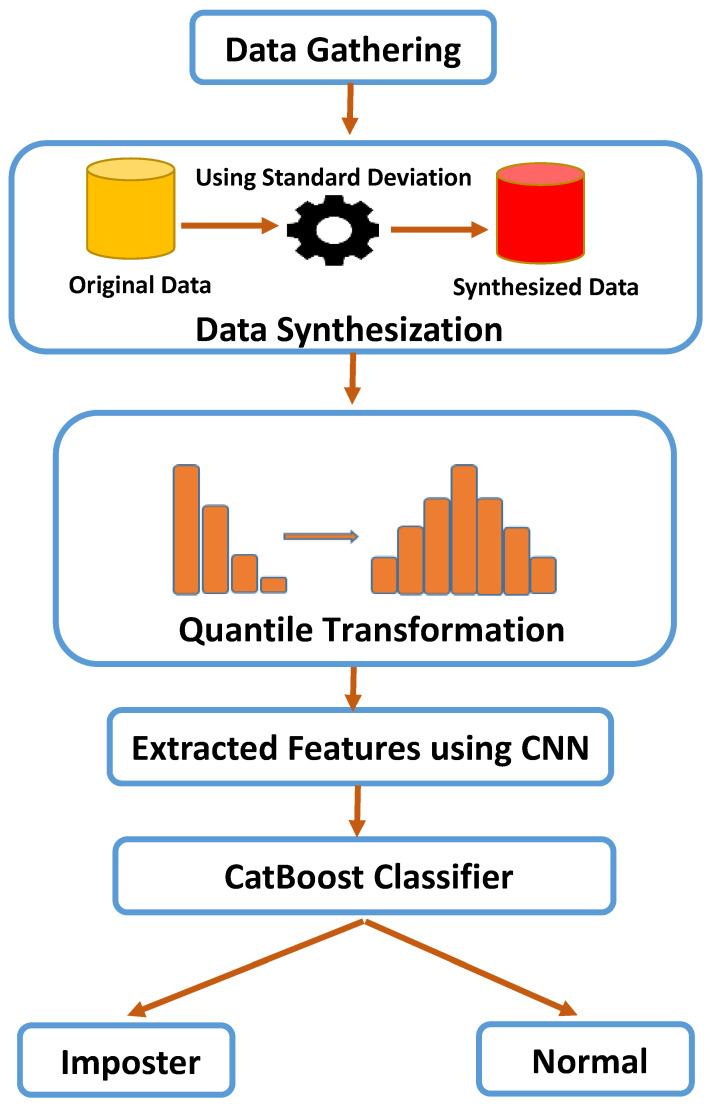
Proposed framework for our methodology.

As illustrated in Figure 1, our approach begins with data gathering that relies on the CMU dataset. This dataset consists of 51 participants who entered their passwords 400 times. Every example contains 31 features. The number of examples for each user is increased via data synthesization, which is important and necessary to enhance model performance. After data synthesization, quantile transformation is employed to convert the data distribution into a uniform distribution, which dramatically minimizes the extreme values that impede model performance. These converted features are fed into a robust and efficient convolutional neural network architecture that is designed to extract robust and immutable features, mitigating issues such as overfitting and underfitting. These extracted features are finally fed into ensemble learning algorithms to detect whether the user is normal or malignant. The methods and techniques used in our research, such as preprocessing, CNN architecture, and learning algorithms, are fully described in the remainder of this section.

### 3.1. Preprocessing

**Figure 2:**
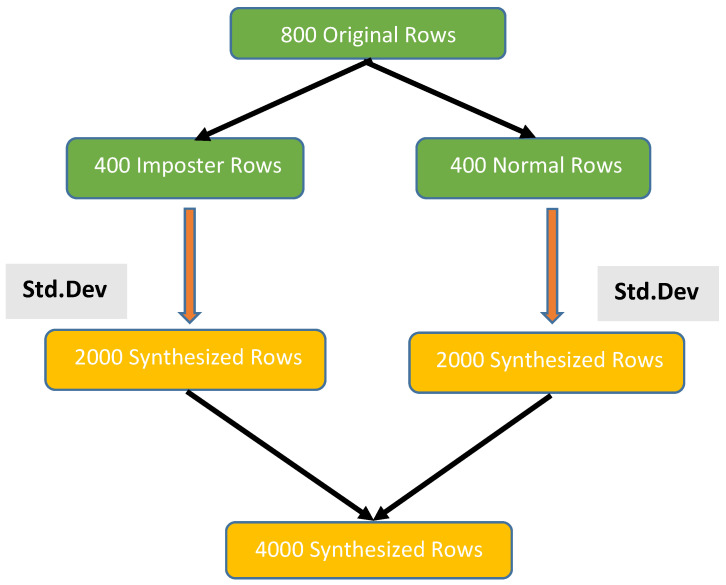
Flow of data synthesization.

- **Data Synthesization (DS)** is considered one of the most important phases in our approach. It generates new synthesized data-based statistical techniques. The standard deviation is utilized to generate new data by computing it for each column feature and then adding it to the old values of the column feature. For every user, there are 800 original rows (400 normal and 400 imposters). Data synthesization is used to generate 5 synthesized rows for each original row, resulting in 2000 normal rows and 2000 imposter rows, with a total of 4000 synthesized rows. Figure 2 illustrates the flow of data synthesization.

- **Quantile Transformation (QT)** is a creative approach that alters the features to achieve a normal distribution. Each feature is individually subjected to this transformation. The native features are converted into novel features that are uniformly distributed using the cumulative distribution function [28]:
  (1) $$F\left(X\right)=\frac{x-a}{b-a}$$
  where *a* and *b* are two fixed values such that *a* <x<b.
  This method is very useful and successful at removing anomalies and extremes, which can have a significant impact on performance.

### 3.2. CNN Architecture

**Figure 3:**
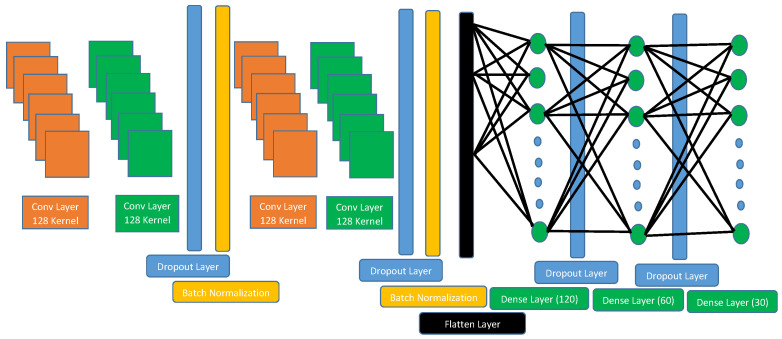
CNN architecture.

As shown in Figure 3, our CNN architecture consists of 14 layers comprising 4 convolutional layers, 4 dropout layers, 2 batch normalizations, 1 flatten layer, and 3 dense layers. In the remainder of this subsection, the important layers of our architecture are explained in detail, including the dropout layer, batch normalization, and ELU activation function.

- **Dropout Layer:** This is a very important technique used to enhance deep learning layers and prevent overfitting problems. It works by shutting down or freezing some neurons according to the dropout rate while unfreezing other neurons. It sets 0 for certain neurons at a specific rate while other neurons are updated by multiplying their values by 1/(1-rate), which ensures that the total sum of all the inputs remains the same before applying dropout [29]. In our architecture, four dropout layers with a rate of 0.4 are used to mitigate the overfitting problem and enable the model to learn different features from a different perspective.
- **Batch Normalization:** Deep learning takes a long time in training due to the different distributions of the batches in each layer. Batch normalization normalizes all the batches of the different distributions into a standard distribution, with the mean set to 0 and the variance set to 1. Then, it scales the inputs and shifts them to another space [30]. The following equations show how batch normalization works.
  (2) $$\mu_{B}=\frac{1}{m}\sum_{i=1}^{m}x_{i}$$
  (3) $$\sigma_{B}^{2}=\frac{1}{m}\sum_{i=1}^{m}{\left(x_{i}-\mu_{B}\right)}^{2}$$
  (4) $${\hat{x}}_{i}=\frac{x_{i}-\mu_{B}}{\sqrt{\sigma_{B}^{2}+\epsilon }}$$
  (5) $$y_{i}=\gamma \hat{x_{i}}+\beta$$
  The mean and variance of the batch are computed, as shown in Equations (2) and (3). Every sample xi of the batch is normalized into a zero mean and unit variance. In Equation (5), the samples are scaled and shifted with learnable parameters γ and β. In our architecture, we use two batch normalization layers, which enhances our approach and has a significant effect on preventing the overfitting problem.
- **ELU Activation Functions:** The Exponential Linear Unit (ELU) is a modified and developed version of the RELU function that suffers from the dying problem. The RELU function works by passing the positive values while setting the negative values to zero. The network does not learn anything during the backpropagation due to the zero output for the negative values. This problem is called the dying problem. The ELU function solves this problem using the following equation [31]:
  (6) $$f\left(x\right)=\left\{\begin{array}{ccc} \alpha \left(e^{x}-1\right) & \;\mathrm{for}\; & x<0\\ x & \;\mathrm{for}\; & x\geq 0 \end{array}\right.$$

The positive values are passed while the negative values are smoothed by the α constant. The ELU has fast convergence and better generalization than the RELU. In addition, it avoids the problems of vanishing or exploding gradients. The ELU function is utilized in our architecture through the convolutional and dense layers. The ELU efficiently solves the dying problem caused by the RELU function and enhances model performance.

Table 2 presents the details of our CNN architecture and the learned parameters for each layer.

### 3.3. Learning Algorithms

In our study, a variety of ensemble learning algorithms that offer reduced training time and improved efficiency are examined. These algorithms belong to the boosting method which is a branch of ensemble learning. The boosting method consists of several algorithms that turn weak learners into strong ones. It is very popular, efficient, and produces outstanding results. The algorithms are described in this subsection.

- **LightGBM:** Gradient Boosting Decision Tree (GBDT) is time-consuming and has low efficiency, especially in a large and high-dimensional dataset, as it scans all the features to calculate the information gain for each potential split. LightBoost is an extension and improvement of GBDT [32] that has proven to function effectively and extremely fast on big datasets and requires significantly less training time than the other algorithms.
- **XGBoost** is exclusively designed and optimized for model effectiveness and computational speed. It makes full use of each byte of memory and hardware and offers the advantages of algorithm improvement, model tuning, and deployment in computing settings. XGBoost enhances performance by optimizing the objective function, as shown in the following equation [33]:
  (7) obj(θ)=TL(θ)+R(θ)
  The objective function consists of two terms (*TL* and *R*). *TL* refers to the training loss that computes the difference between the prediction and actual labels and *R* refers to the regularization that penalizes the training loss in order to solve the overfitting problem and makes the model generalize efficiently on the unseen data.
- **AdaBoost:** The AdaBoost model of Freund and Schapire [34] was the first useful boosting model and is now one of the most popular and extensively studied models, with implementations in many different industries. The AdaBoost technique is built on the concept of merging numerous weak rules to obtain a high-accuracy prediction rule. The sign of a weighted aggregation of weak classifiers is computed by the final or combined classifier *F* [35].
  (8) $$F\left(x\right)=\sum_{t=1}^{T}\alpha_{t}h_{t}\left(x\right)$$
  where the final classifier is computed as a weighted majority vote of the weak classifiers $h_{t}$, where each classifier is given the weight $\alpha_{t}$.
  AdaBoost aims to minimize the cost function, also known as the exponential loss [35]
  (9) $$\frac{1}{m}\sum_{i=1}^{m}\exp\left(-y_{i}F\left(x_{i}\right)\right)$$
  where F(x) is as given in Equation (8) and *y* is the actual label.
- **CatBoost** is an open source library that utilizes gradient boosting on decision trees. It is a machine learning algorithm that produces high-quality predictions by using categorical features to build an ensemble of decision trees. CatBoost uses a novel technique called “Ordered Boosting”, which helps to reduce overfitting and improve accuracy. The equation for CatBoost is as follows [36]:
  (10) $$F\left(x\right)=\sum_{i=1}^{N}\alpha_{i}F_{i}\left(x\right)$$
  where $\alpha_{i}$ is the weight of each tree in the ensemble, $F_{i}\left(x\right)$ is the prediction of the *i*th tree, and *x* is the input vector.

We utilized a variety of software tools in our implementation, including Pandas for data processing; Matplotlib, Matlab, and Python for visualization; Sklearn library for machine learning algorithms; and Keras and TensorFlow for the convolutional neural network.

## 4. Experiments and Results

### 4.1. Dataset

**Figure 4:**
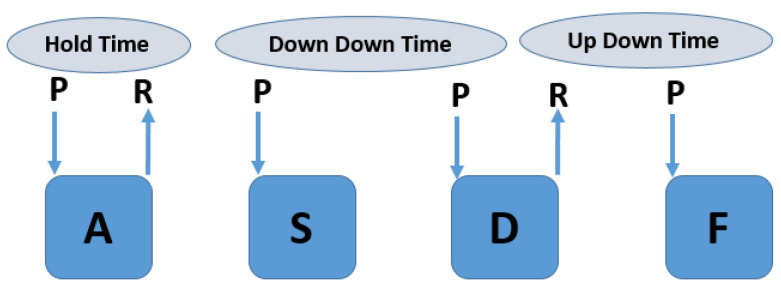
Features of keystroke dynamics.

The CMU dataset consists of 51 users’ keyboard dynamics. Each user inputs the password “.tie5Roanl” 400 times in 8 sessions, with 50 repetitions per session. The sessions were spaced at least one day apart to capture daily variations in rhythm [37]. The model was given various timing features as input, including Hold Time, Down-Down Time, and Up-Down Time features. Hold Time describes the duration between pressing and releasing a key. Down-Down Time represents the time elapsed between two consecutive key presses, and Up-Down Time describes the time elapsed between releasing a key and pressing the next key. The CMU dataset includes a total of 31 timing features, with 11 categorized as Hold-Time, 10 as Down-Down Time, and 10 as Up-Down Time features. The timing features used in the model are presented in Figure 4. A key press is represented by a down arrow and a key release is represented by an up arrow, as described in [22].

### 4.2. Evaluation

**Figure 5:**
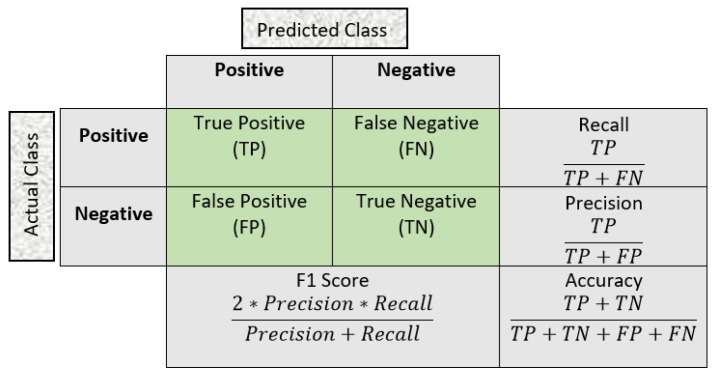
Confusion matrix and evaluation metrics.

Many previous studies used common evaluation metrics such as accuracy and EER. However, in our study, we incorporated additional evaluation metrics, such as precision, recall, and F1 score, which are shown in Figure 5. The ROC curve was utilized to create a graphical representation of the model’s performance and robustness, and the area under the curve (AUC) was computed to determine the quality of the classifier. An AUC of 1 indicates a perfect classifier, whereas an AUC of 0.5 indicates a random classifier. The EER was calculated using the ROC curve by determining the point where the rates of false acceptance and false rejection were equal, as described in [38]. The lower the EER, the better the performance of the algorithm. Our study considered the use of multiple evaluation metrics to ensure that our methodology is efficient, robust, and free from overfitting and underfitting problems.

### 4.3. Data Exploration

The CMU dataset contains 31 features divided into 3 subsets: Down-Down Time, Up-Down Time, and Hold Time features. We performed a statistical analysis to determine whether there were significant differences between the three subsets. To conduct data exploration, 4 out of 51 users were randomly selected.

**Figure 6:**
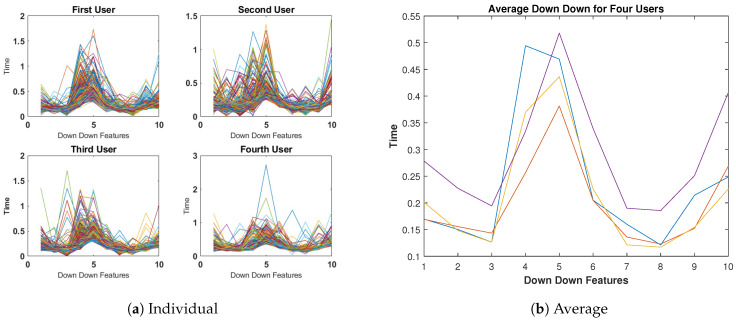
Down-Down Time features for four subjects (400 keystrokes).

The line graphs presented in Figure 6a show 400 input samples for each user, and the patterns in these samples indicate a significant level of consistency in the Down-Down Time features subset. This suggests that users can be accurately classified based on their typing patterns. Furthermore, by comparing the average cases of the four selected users in Figure 6b, it can be observed that their typing patterns were quite similar to each other.

**Figure 7:**
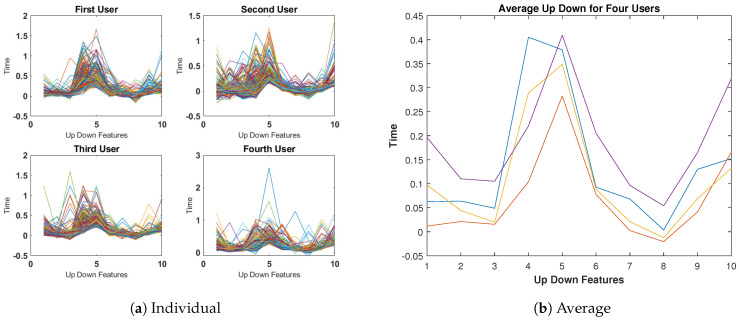
Up-Down Time features for four subjects (400 keystrokes).

The outcomes for the Up-Down Time features are displayed in Figure 7, which appear to be similar to those of the Down-Down Time features illustrated in Figure 6.

**Figure 8:**
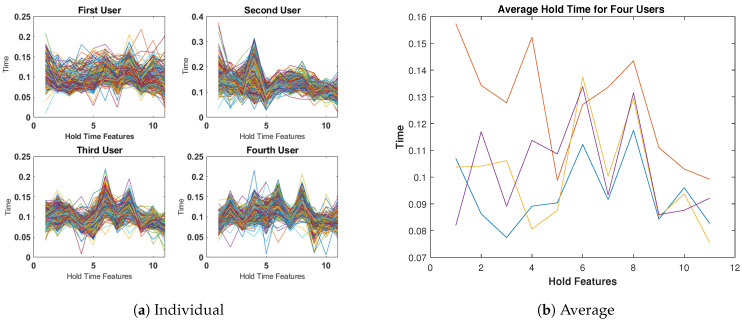
Hold-Time features for four subjects (400 keystrokes).

Figure 8a compares the four users based on the Hold-Time features, where it is more apparent that there are variations among them. The average examples in Figure 8b show even more significant differences. Therefore, these observations suggest that the Hold-Time features can be used to distinguish between users.

### 4.4. Quantile Transformation Effect

**Figure 9:**
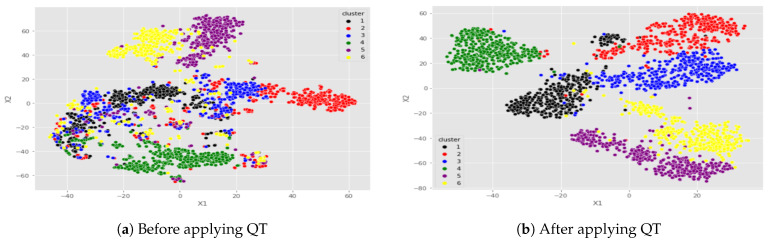
Projection of 6 users’ features before and after applying QT.

Figure 9 depicts the features of six users and demonstrates the importance of QT. For each user, 400 samples were regarded, and their features were retrieved and represented utilizing the t-SNE mechanism [39]. The same features are presented from two perspectives: in Figure 9a, before applying QT, and in Figure 9b, after applying QT. The utilization of QT in Figure 9b helps in narrowing the gap between users’ anomalies and non-anomalies, making it simpler for our approach to recognize users. Although the samples from the six users are merged, applying QT allows for the easy differentiation of the correct clusters.

**Figure 10:**
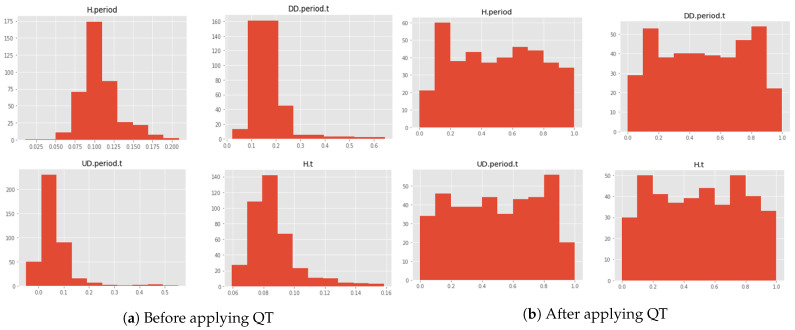
Distribution of 6 users’ features before and after applying QT.

Furthermore, in order to emphasize the significance of QT and illustrate the process of transforming data into a standard normal distribution, Figure 10 presents a visualization of this transformation by selecting 400 samples of 4 random timing features for a specific user. Figure 10a displays the original distribution of the data, whereas Figure 10b displays the variation in the data distribution after applying QT. The transformed data show significantly improved conformity to a standard normal distribution, which effectively minimizes the occurrence of outliers.

### 4.5. Results

In our study, we assessed the effectiveness of our proposed methodology using the CMU Benchmark Dataset. To evaluate the model, we designated 1 user as genuine and 50 as imposters. We randomly selected 400 timing features for the genuine user and 400 timing features for each imposter, with 8 timing features taken from each imposter, resulting in a combined total of 800 features. The dataset was split into training and testing sets, with 70% of the data used for training and the remaining 30% for testing. This was done for each user, with the user being treated as genuine and the other users as imposters. The evaluation process used is described in [34].

To assess the performance and robustness of our approach, we employed efficient boosting algorithms, including LightGBM, XGBoost, AdaBoost, and CatBoost. The results demonstrate that our methodology performs well and is robust.

- **Performance:**

**Figure 11:**
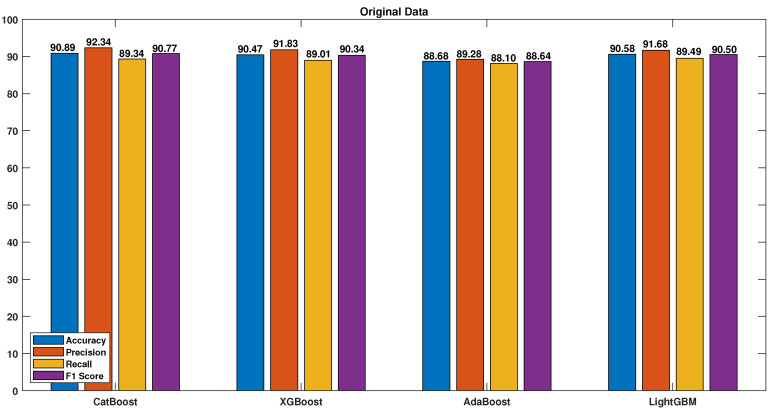
Original data.

Four boosting algorithms were used to evaluate the performance of the models trained on the features extracted from the tailored convolutional neural network. The algorithms were tested with various preprocessing techniques, and the results were measured using accuracy, precision, recall, and F1 score. Figure 11 displays the results obtained when training the boosting algorithms on the original data without any preprocessing techniques, which demonstrated poor performance.

**Figure 12:**
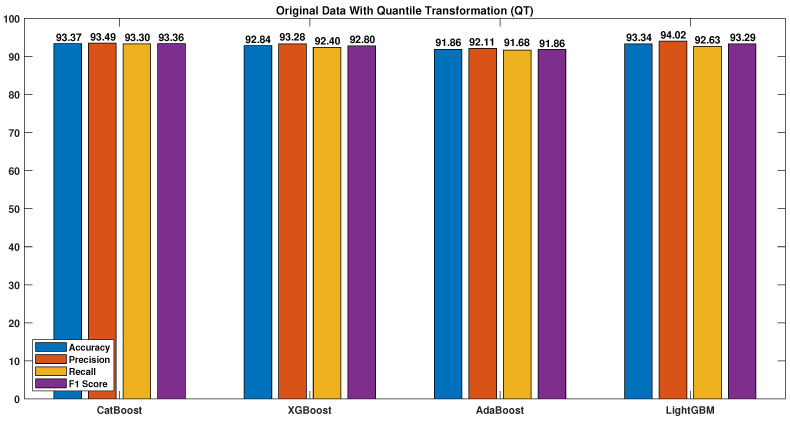
Original data with quantile transformation (QT).

However, by applying quantile transformation, as shown in Figure 12, the results were enhanced by transforming the data into a normal standard distribution, thereby reducing the impact of outliers.

**Figure 13:**
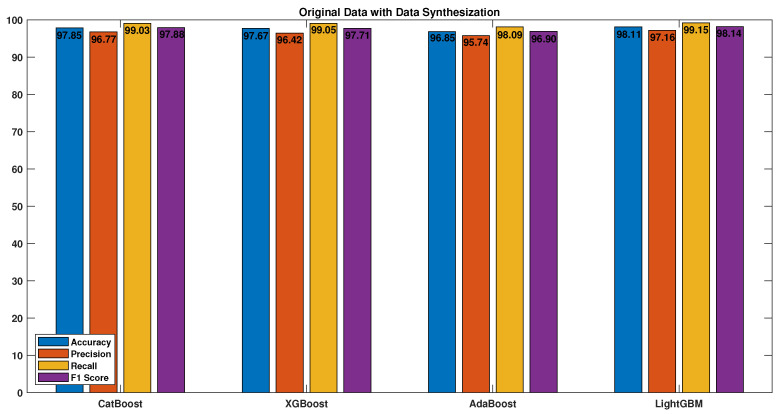
Original data with data synthesization (DS).

According to the results presented in Figure 13, applying data synthesization led to a significant improvement. The performance of our methodology greatly increased when the dataset was increased from 800 to 4000 samples. This expansion of the dataset not only resulted in better performance but also prevented our methodology from experiencing issues related to overfitting and underfitting.

**Figure 14:**
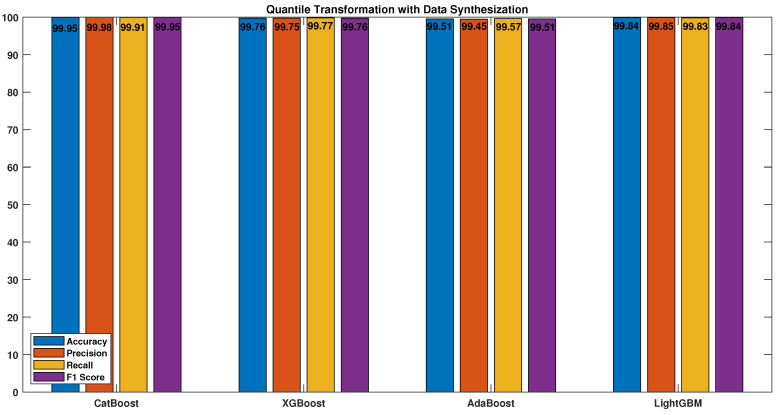
Data synthesization (DS) with quantile transformation (QT).

The boosting algorithms exhibited their highest level of performance when both quantile transformation and data synthesization techniques were combined, as depicted in Figure 14. The results clearly demonstrate that the utilization of these two techniques in tandem is highly beneficial for improving the performance of the boosting algorithms.

**Figure 15:**
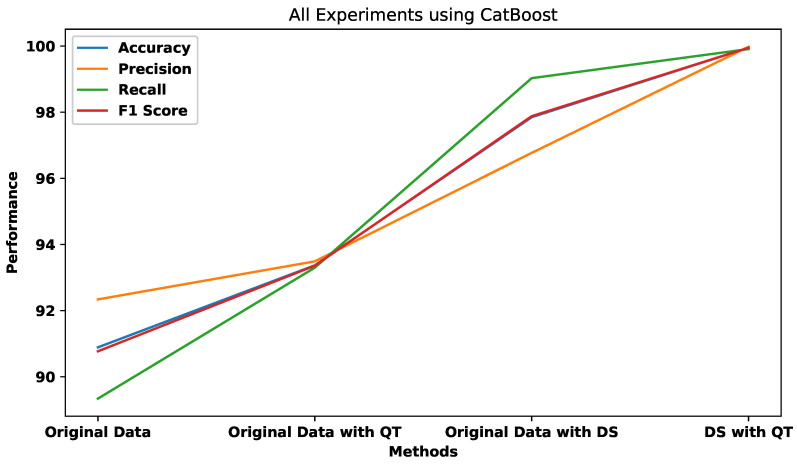
All experiments using CatBoost.

All the algorithms demonstrated significant improvements after applying both quantile transformation (QT) and data synthesization (DS), especially in the case of the CatBoost algorithm. The accuracy, precision, recall, and F1 score of the CatBoost algorithm increased to 99.95, 99.98, 99.91, and 99.95, respectively. The performance of the best-performing classifier (CatBoost) was evaluated using four different sets of data: original data only, original data with QT, original data with DS, and QT with DS. Figure 15 presents the results, which clearly show that the performance of the classifier was significantly enhanced through the use of various preprocessing techniques.

**Figure 16:**
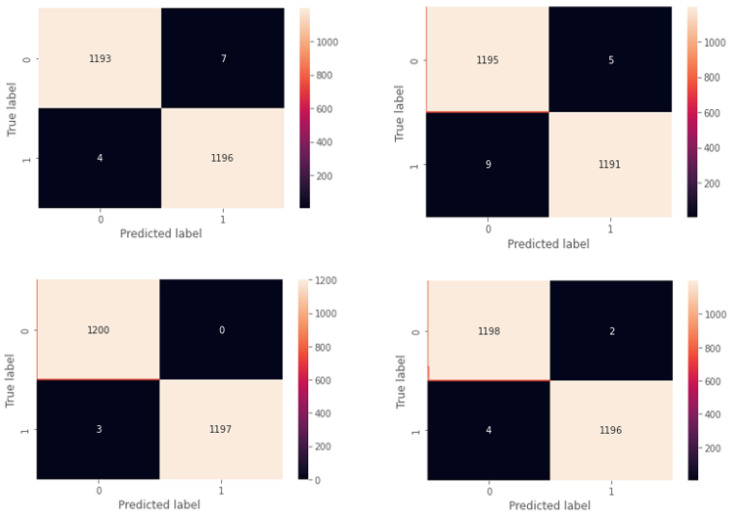
Confusion matrix for four random users.

Figure 16 depicts the performance of the CatBoost algorithm in the form of a confusion matrix. To showcase the effectiveness of the model, we randomly selected four users and computed their confusion matrices. The results indicated that the model could accurately classify almost all the samples for each user, with only a few instances of misclassification.

Table 3 presents a summary of the results obtained from the evaluation of all the preprocessing techniques and boosting algorithms.

The table highlights the importance of the quantile transformation and data synthesization techniques and shows the results before and after applying both techniques to all the models. Applying DS and QT led to significant improvements for all the models, especially the CatBoost algorithm, which achieved accuracy, precision, recall, F1 score, and EER values of 99.95, 99.98, 99.91, 99.95, and 0.65, respectively, compared to values of 90.89, 92.34, 89.34, 90.77, and 7.6 without DS and QT.

- **Robustness:**

One of the key aspects of evaluating the effectiveness and reliability of any new technique or approach is conducting a rigorous analysis of its performance metrics. In this regard, the ROC curve is a widely recognized and highly effective tool that can be used to assess the robustness and efficiency of a given approach.

**Figure 17:**
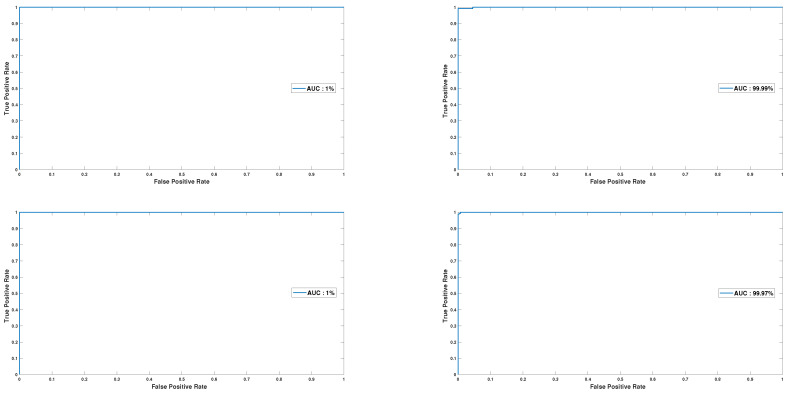
ROC curves for four random users.

To demonstrate the effectiveness of our approach, we utilized the ROC curve as a metric to evaluate its performance. In particular, we randomly selected four users and generated ROC curves for each of them, which are illustrated in Figure 17.

The results indicate a significant improvement in the performance of our approach, with the curves approaching a value of 1. This suggests that our approach is highly robust, efficient, and capable of delivering accurate results.

**Figure 18:**
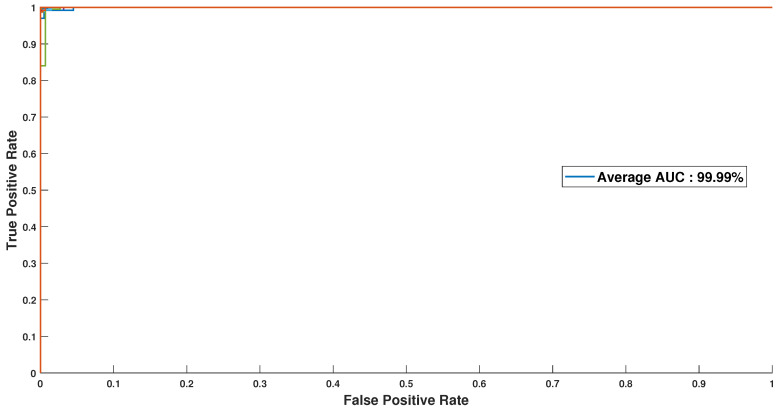
ROC curves for 51 users.

In order to further reinforce the robustness and efficiency of our approach, we visualized the ROC curves for 51 users, as shown in Figure 18, where it can be seen that the plotted curves consistently approach the upper boundary, indicating that our approach is highly reliable and consistent across a large and diverse user base. The average AUC (Area Under the Curve) value for the 51 ROC curves was found to be 99.99%, which is a strong indicator of the success and effectiveness of our methodology.

Overall, the ROC curve analysis provides a powerful and highly informative tool for assessing the performance of our approach. The results clearly demonstrate that our approach is not only highly robust and efficient but also highly reliable and consistent across a large user base. These findings are likely to have significant implications for a wide range of applications and industries, where accurate and reliable performance metrics are critical for achieving success.

**Figure 19:**
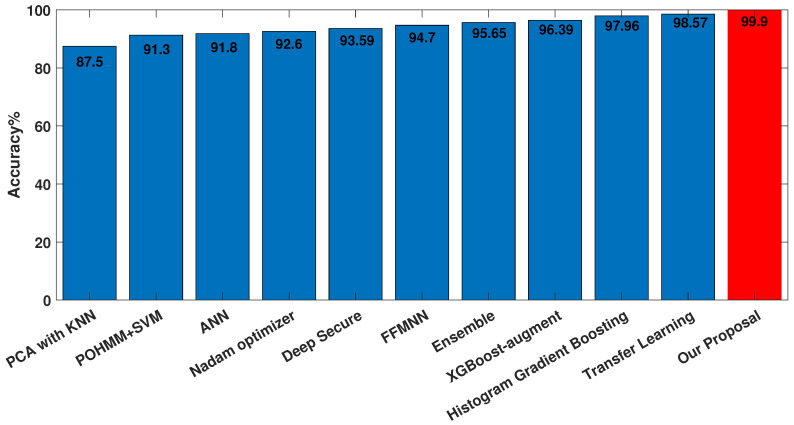
Comparison of accuracy with the related literature (from left to right: [17], [19], [21], [25], [26], [22], [20], [12], [11], [23]).

To benchmark our research against prior literature, we analyzed the evaluation metrics used in previous studies. Our investigation revealed that accuracy and EER were the most commonly used metrics in prior research. Hence, we adopted these metrics to compare the performance of our approach with that of earlier studies. As demonstrated in Figure 19, the accuracy of our approach was compared with that of prior studies. The results demonstrate that our model outperformed all of the previous algorithms in terms of accuracy.

**Figure 20:**
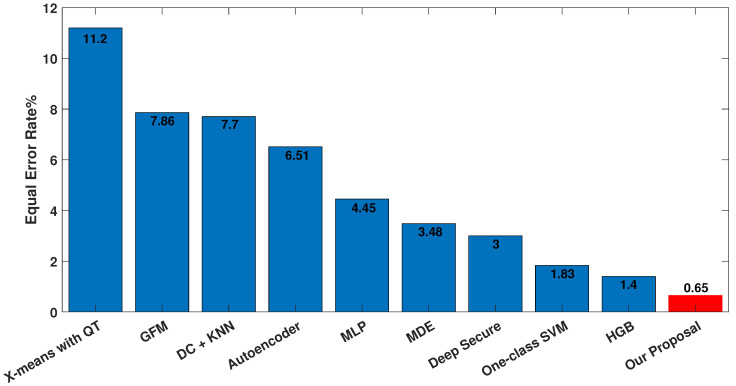
Comparison of EER with the related literature (from left to right: [15], [18], [16], [24], [27], [14], [26], [13], [11]).

Additionally, we compared the EER of our model with that of prior studies, as shown in Figure 20. Notably, our approach achieved the lowest EER among all the algorithms previously proposed in the literature. These findings further underscore the superior performance and efficacy of our proposed approach in comparison to earlier methods.

## 5. Conclusions

This research introduces a tailored convolutional neural network that is specifically designed for feature extraction, as well as a boosting technique to improve classification accuracy in keystroke dynamics-based user authentication. The proposed model addresses the challenges of lower accuracy and robustness by utilizing innovative data synthesization techniques and quantile transformation methods. The approach demonstrates excellent performance, achieving an average accuracy of 99.95%, an average equal error rate of 0.65%, and an average area under the curve of 1% on the CMU dataset, surpassing recent developments in this field. This study contributes to the development of user authentication systems and can improve the security of online services. Future research will focus on using transfer learning approaches, autoencoders, graph neural networks, and generative adversarial networks (GANs) to further advance the model.

## Figures and Tables

**Table 1:** Latest developments in the CMU dataset.

Reference	Classifier	Date	Accuracy (%)	EER (%)
[11]	Histogram Gradient Boosting	2023	97.96	1.4
[12]	XGBoost-augment	2022	96.39	-
[13]	One-class SVM	2021	-	1.83
[14]	MDE	2019	-	3.48
[15]	X-means with QT	2021	AUC is 0.942	11.2
[16]	Dependence Clustering + KNN	2017	-	7.7
[17]	Kernel PCA with KNN	2020	87.5	-
[18]	GFM	2018	-	7.86
[19]	POHMM/SVM	2022	91.3	-
[20]	Ensemble (KNN, SVM, DT)	2022	95.65	-
[21]	ANN	2021	91.8	-
[22]	FFMNN	2020	94.7	-
[23]	Transfer Learning	2022	98.57	-
[24]	Autoencoder	2019	-	6.51
[25]	Nadam optimizer	2018	92.60	-
[26]	Deep Secure	2017	93.59	3
[27]	MLP	2020	-	4.45

**Table 2:** Details of all layers in CNN architecture.

Layer Type	Kernel Size	Padding	Activation Function	# of Kernels	Output Shape	Learned Parameters
Conv 1D	7 × 1	Same	Elu	128	31 × 128	1024
Conv 1D	7 × 1	Same	Elu	128	31 × 128	114,816
Dropout	-	Rate = 0.4	-	-	31 × 128	0
BatchNormalization	-	-	-	-	31 × 128	512
Conv 1D	5 × 1	Same	Elu	64	31 × 64	41,024
Conv 1D	5 × 1	Same	Elu	64	31 × 64	20,544
Dropout	-	Rate = 0.4	-	-	31 × 64	0
BatchNormalization	-	-	-	-	31 × 64	256
Flatten	-	-	-	-	1984	0
Dense (120)	-	-	Elu	-	120	238,200
Dropout	-	Rate = 0.4	-	-	120	0
Dense (60)	-	-	Elu	-	60	7260
Dropout	-	Rate = 0.4	-	-	60	0
Dense (30)	-	-	Elu	-	30	1830

**Table 3:** The impact of data synthesization (DS) and quantile transformation (QT).

Method/Metric		Accuracy (%)	Precision (%)	Recall (%)	F1 Score (%)	ERR (%)
**DS + QT (Off)**	**LightGBM**	90.58	91.68	89.49	90.50	8.2
	**XGBoost**	90.47	91.83	89.01	90.34	8.5
	**AdaBoost**	88.68	89.28	88.10	88.64	11.2
	**CatBoost**	90.89	92.34	89.34	90.77	7.6
**DS + QT (On)**	**LightGBM**	99.84	99.85	99.83	99.84	0.15
	**XGBoost**	99.76	99.75	99.77	99.76	0.24
	**AdaBoost**	99.51	99.45	99.57	99.51	0.53
	**CatBoost**	**99.95**	**99.98**	**99.91**	**99.95**	**0.65**

## Data Availability

This study did not involve the creation of any new data. The dataset is publicly available at https://www.cs.cmu.edu/~keystroke/ (accessed on 3 January 2022).
